# Ionizable lipid nanoparticles of mRNA vaccines elicit NF-κB and IRF responses through toll-like receptor 4

**DOI:** 10.1038/s41541-025-01124-x

**Published:** 2025-04-17

**Authors:** Amanda E. Zelkoski, Zhongyan Lu, Gauthaman Sukumar, Clifton Dalgard, Hooda Said, Mohamad-Gabriel Alameh, Edward Mitre, Allison M. W. Malloy

**Affiliations:** 1 Department of Pediatrics, Uniformed Services University of Health Sciences, Bethesda, MD USA; 2 Henry M Jackson Foundation for the Advancement of Military Medicine, Bethesda, MD USA; 3 Department of Anatomy, Physiology & Genetics, Uniformed Services University of Health Sciences, Bethesda, MD USA; 4 Department of Pathology and Laboratory Medicine, Children’s Hospital of Philadelphia, Philadelphia, PA USA; 5 Department of Pathology and Laboratory Medicine, University of Pennsylvania, Philadelphia, PA USA; 6 Penn Institute for RNA Innovation, University of Pennsylvania, Philadelphia, PA USA; 7 Department of Microbiology and Immunology, Uniformed Services University of Health Sciences, Bethesda, PA USA

**Keywords:** Adjuvants, Toll-like receptors, RNA vaccines

## Abstract

Ionizable lipid nanoparticles (LNP) that have enabled the success of messenger RNA (mRNA) vaccines have been shown to be immunostimulatory in the absence of mRNA. However, the mechanisms through which they activate innate immune cells is incompletely understood. Using a monocyte cell line, we compared the ability of three LNP formulations to activate transcription factors Nuclear Factor-kappa B (NF-κB) and Interferon Regulatory Factor (IRF). Comparison of signaling in knockout cell lines illustrated a role for Toll-like receptor (TLR) 4 in initiation of this signaling cascade and the contribution of the ionizable lipid component. Activation induced by empty LNPs was similar to that induced by LNPs containing mRNA, indicating that LNPs may provide the majority of innate stimulation for the mRNA vaccine platform. Our findings demonstrate that ionizable lipids within LNPs signal through TLR4 to activate NF-κB and IRF, identifying a mechanism for innate activation that can be optimized for adjuvant design.

## Introduction

Nanoparticles have been developed for use in the delivery of therapeutics and vaccine antigen. Distinct lipid nanoparticle (LNP) formulations were used for the BNT162b2 and mRNA-1273 vaccines to deliver a N1-methylpseudouridine-modified mRNA encoding SARS-CoV-2 spike^1,2^. Despite demonstrated immunogenicity, durability is limited^3–8^. Furthermore, the cause of the observed side effect profile is unknown^9,10^. These findings present an opportunity for enhancing both tolerability and efficacy. However, achieving this improvement requires a deeper understanding of the functional mechanisms of mRNA-LNP vaccines. How these vaccines initiate immune responses is incompletely understood. Vaccines typically contain an adjuvant that stimulates the immune response, and some elicit this response through Toll-like receptor (TLR) signaling^11,12^. The mRNA component may signal through TLR7 and 8, which recognize single-stranded RNA (ssRNA). Additionally, double-stranded RNA (dsRNA), a byproduct of the mRNA production process, may elicit signaling through TLR3, RIG-I, or MDA-5. However, the mRNA incorporated into these vaccines have undergone modifications aimed at diminishing ssRNA recognition and been purified to reduce dsRNA presence^1,7,13–16^. Interestingly, both in vitro and in vivo studies have demonstrated that the empty LNP can activate the innate immune system^17–19^. However, the mechanisms behind LNP-mediated immunostimulation are poorly understood.

LNPs vary in composition, but are primarily designed with a charged or ionizable lipid and helper lipids^20^. The LNP of the BNT162b2 mRNA vaccine is comprised of the ALC-0315 ionizable cationic lipid, cholesterol, DSPC (1,2-distearoyl-sn-glycero-3-phosphocholine), and ALC-0159, a PEGylated lipid^1,21^. In comparison, the LNP of mRNA-1273 uses SM102 as the ionizable lipid and PEG-DMG as the PEGylated lipid^1,21,22^. The ionizable lipids are neutral at time of administration but become positively charged in the acidic endosome, which facilitates LNP fusion and mRNA release into the cytoplasm^23^. Cholesterol and PEGylated helper lipids prevent aggregation or enhance transfection efficiency^1,20^. The components also impact size, with the LNPs of the BNT162b2 and mRNA-1273 vaccines being 90.2 ± 7.8 nm and 75.5 ± 0.4 nm, respectively^22^. A review of LNP formulations found that 61% elicited an immune response, with complement and innate activation predominating^24–26^. Some cationic LNPs can be detected by TLR4 and the NLRP3 inflammasome^25,27–31^. Additionally, antibodies generated to the PEGylated lipid can activate the complement pathway^32^. It is unclear whether empty ionizable LNPs would activate the immune system through similar pathways as cationic formulations since the vaccines incorporate distinct ionizable cationic lipids that may differentially influence uptake and degradation^31,33^.

Recently, LNP, in the absence of mRNA, was demonstrated to be immunostimulatory in a murine model^18^. Removal of the ionizable lipid in this LNP resulted in a reduction in cytokine production, suggesting the ionizable lipid plays an important role in adjuvanticity^18^. Similarly, stimulation of human monocyte-derived dendritic cells with ionizable LNPs elicits production of cytokines such as IL-1, IL-6, IL-12, IFNα, and IFNγ^17,19^. To date, however, the mechanisms by which empty LNPs activate the innate immune system are not well understood.

Alameh et al. observed that the adjuvanticity of an empty ionizable LNP in a murine model was partially reliant on MyD88, suggesting a role for TLRs or IL-1 receptors^18,34^. TLRs can activate nuclear factor ĸB (NF-κB) and interferon regulatory factor (IRF) transcription factors through MyD88 and TRIF adaptor molecules^35^. These transcription factors are essential to the innate response as they regulate the production of cytokines, chemokines, and type I interferons (IFNs)^35^. However, others have found no evidence for TLR involvement in the innate immune response to LNPs in mouse models^36^.

In this study, we sought to investigate the ability of empty ionizable LNPs to activate human innate immune cells. Therefore, we compared innate immune activation induced by three LNPs using a human monocyte cell line. We determined that ionizable LNPs differentially activate NF-κB and IRF. Our findings show signaling is predominantly mediated through TLR4. Interestingly, NF-κB and IRF responses to the empty BNT162b2 LNP were similar in magnitude to mRNA-LNP, indicating that the ionizable LNP may be primarily responsible for activating the innate immune response during mRNA vaccination.

## Results

### The ionizable LNPs elicits NF-κB and IRF responses from THP-1 cells

Monocytes are key innate immune cells that rapidly respond to pathogen or damage-associated stimuli. Activation and function of monocytes is associated with NF-κB activity and IRF pathway activation^37,38^. To determine if empty ionizable LNPs could activate monocytes, we utilized a THP-1 monocyte-like cell line with an alkaline phosphatase reporter for NF-κB activation and a luciferase reporter for IRF activation. We compared activation induced by different ionizable LNPs to R848 (TLR7/8 agonist), and MPLA (TLR4 agonist). The empty LNPs used were LNP-1, LNP-ALC315 (BNT162b2 formulation), and LNP-SM102 (mRNA-1273 formulation) (Supplementary Table 1). LNP-SM102 differs in ionizable lipid structure from LNP-ALC315 as well as in PEGylated lipid (Supplementary Fig. 1) (Supplementary Table 1).

**Fig. 1:**
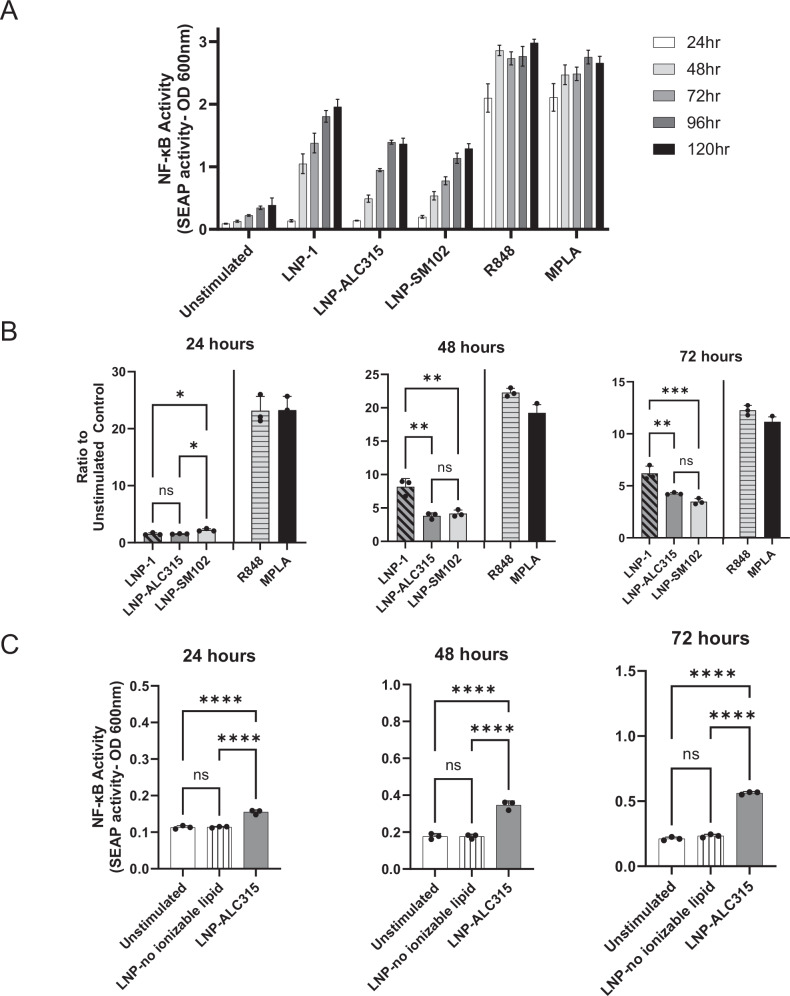
NF-κB signaling transduction in THP-1 reporter cell line. THP-1-Dual reporter cells were treated with LNP-1 (1 μg/mL), LNP-ALC315 (1 μg/mL), LNP-SM102 (1 μg/mL), R848 (1 μg/mL), MPLA (1 μg/mL), LNP without ionizable lipid (1 μg/mL), or media alone. An alkaline phosphatase reporter was used to measure the NF-κB response. Reporter accumulation was measured in the supernatant over 120 h (*n* = 3) (**A**). Fold change reporter production was compared between LNPs (*n* = 3) (**B**). Production of reporter in response to LNP without ionizable lipid was compared to the compositionally similar LNP-ALC315 (*n* = 3) (**C**). Data are represented as mean ± SEM. Significance was assessed using a one-way ANOVA with Dunnett’s test for multiple comparisons. *P ≤ 0.05, ** *P* ≤ 0.01, ***P ≤ 0.001, ****P ≤ 0.0001.

Stimulation of THP-1s resulted in NF-κB reporter detection at 24 h, with increased NF-κB activation at 48 h that minimally increased or plateaued through 120 h (Fig. 1A). For LNP-ALC315, the NF-κB response was 2-fold above the unstimulated control at 24 h and then 4-fold past 48 h. The response to LNP-1 was 6–7-fold at 48 and 72 h. The response to LNP-1 was 6–7-fold at 48 and 72 h. NF-κB activation induced by LNP-SM102 was similar in magnitude to that of LNP-ALC315 (Fig. 1). The kinetics of reporter production to all LNPs increased over 24 h with activation peaking at 48 and 72 h (Supplementary Fig. 3A). In comparison, all TLR agonists exhibited strong responses within 24 h. We also observed that NF-κB signal transduction was dose dependent and plateaued at the highest dose regardless of the LNP (Supplementary Fig. 2A). All conditions exhibited similar changes in viability over time cultured, and R848 and MPLA exhibited the highest percentage of cell death (Supplementary Fig. 3C). As decrease of viability is seen in all conditions, there is the potential for cell death products to contribute to activation seen, particularly as cell death begins around the initiation of NF-κB detection. At 48 h, LNP-1 induces the greatest levels of cell death corresponding with the heightened NF-κB activation compared to the other LNP formulations, while LNP-SM102 stimulation induces similar levels of death with minimal NF-κB activation at this time point.

To investigate the cause of the innate activation, we utilized a LNP that lacks the ionizable lipid component to determine the contribution to stimulation. This LNP is comprised of cholesterol, DSPC, and ALC-0159, the same composition as LNP-ALC315 except devoid of ionizable lipid (Supplementary Table 1). The NF-κB response was lost in the absence of the ionizable lipid component (Fig. 1C) indicating the primary contribution of the ionizable lipid to the NF-κB activation.

**Fig. 2:**
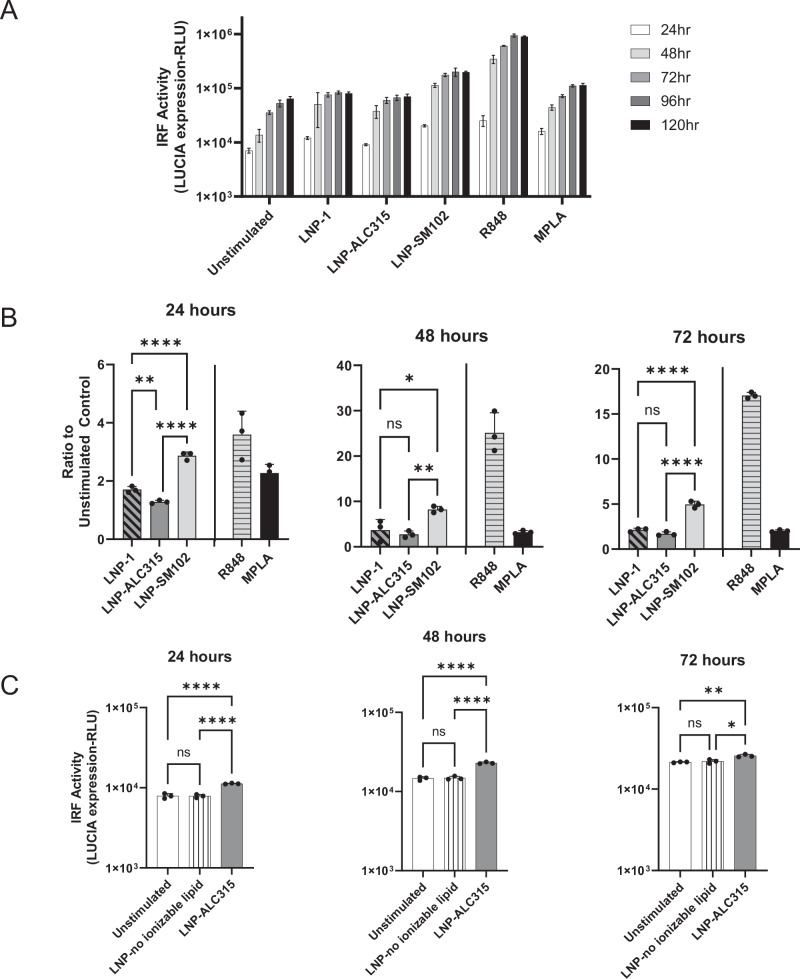
IRF signaling transduction in THP-1 reporter cell line. THP-1-Dual reporter cells were treated with LNP-1 (1 μg/mL), LNP-ALC315 (1 μg/mL), LNP-SM102 (1 μg/mL), R848 (1 μg/mL), MPLA (1 μg/mL), LNP without ionizable lipid (1 μg/mL), or media alone. A luciferase reporter was used to measure the IRF response. Reporter accumulation was measured in the supernatant over 120 h (*n* = 3) (**A**). Fold change reporter production was compared between LNPs (*n* = 3) (**B**). Production of reporter in response to LNP without ionizable lipid was compared to the compositionally similar LNP-ALC315 (*n*= 3) (**C**). Data are represented as mean ± SEM. Significance was assessed using a one-way ANOVA with Dunnett’s test for multiple comparisons. *P ≤ 0.05, **P ≤ 0.01, ***P ≤ 0.001, ***P ≤ 0.0001.

We also investigated the activation of IRF pathways in response to the ionizable LNPs. We measured a 3 and 1.7-fold increase at 48 and 72 h, respectively, in the IRF reporter in response to LNP-ALC315 in comparison to the control (Fig. 2A, B). Unlike with NF-κB, LNP-1 had a similar IRF response to LNP-ALC315 while LNP-SM102 elicited a significantly greater response (Fig. 2A, B). The TLR agonist R848 exhibited the highest increase in IRF response, which was significantly higher than the empty LNPs and TLR agonist MPLA (Supplementary Fig. 3B). We also observed a dose dependent IRF response to each LNP that plateaued at 2 µg/ml (Supplementary Fig. 2B). Additionally, we assessed the ionizable lipid’s contribution and showed that an LNP lacking ionizable lipid was unable to elicit IRF activation (Fig. 2C). This indicates that the IRF response, like NF-κB, is primarily driven by the ionizable lipid component.

Overall, our data show empty ionizable LNPs predominantly activate NF-κB and, to a lesser extent, IRF transcription factors. The magnitude of these responses differs between the ionizable LNPs, but, importantly, was lost in the absence of the ionizable component.

### LNP endocytosis is not associated with transcription factor activation

Signaling cascades, once initiated, typically result in NF-κB and IRF phosphorylation in a short time period, even as quickly as 1 h^39,40^. The activation of the NF-κB and IRF pathways by ionizable LNPs peaked at 48–72 h after stimulation, which was delayed compared to activation by the TLR agonists. To address the slower response kinetics, we first confirmed it was consistent at different doses (Supplementary Fig. 2). We then hypothesized that endocytosis may be required for LNPs to initiate activation. Using a fluorescent DiO-tagged LNP, we investigated the kinetics of uptake by the THP-1 cells. Uptake was visualized within 2 h, peaking in intensity at 48 h (Supplementary Fig. 4A, B). With lower doses, uptake declines more rapidly at 72 h. The decline is likely due to consumption of DiO-LNP in the media as cells replicate, leaving newly replicated cells unable to uptake DiO-LNP (Supplementary Fig. 4A, B). These findings demonstrate a continuous uptake of LNP by THP-1 cells over time.

As this data is suggestive of a threshold of uptake, we utilized endocytosis inhibitors to determine if this process was indeed critical for cellular activation. We treated THP-1 cells with dynasore, cytochalasin D, chloroquine, or methyl-β cyclodextrin 2 h prior to stimulation and every 24 h subsequently in order to inhibit different pathways of endocytosis^41^. The clathrin-mediated endocytosis (CME) inhibitor chloroquine decreased the IRF responses (Supplementary Fig. 4C). Dynasore also inhibits CME but did not impact LNP-mediated activation (Supplementary Fig. 4C). Thus, the decrease in IRF response seen with chloroquine is potentially the result of an off-target effect. Cell death was not a factor as viability for each condition was within 2% of the untreated control throughout. The other inhibitors did not impact or heightened response. Minimal reduction in uptake of DiO-tagged LNP was observed at 24 h and no inhibition was measured at 48 h (Supplementary Fig. 5). The inhibitors reduced NF-κB and IRF responses to MPLA and R848 controls at early time points (Supplementary Fig. 4D). Overall, we observed no significant impact of endocytosis inhibitors on LNP-mediated NF-κB and IRF activation. However, given the limited inhibition visualized, we cannot definitively conclude that activation does not require endosomal entry.

### Stimulation with ionizable LNPs elicit upregulation of toll-like receptor signaling

**Fig. 3:**
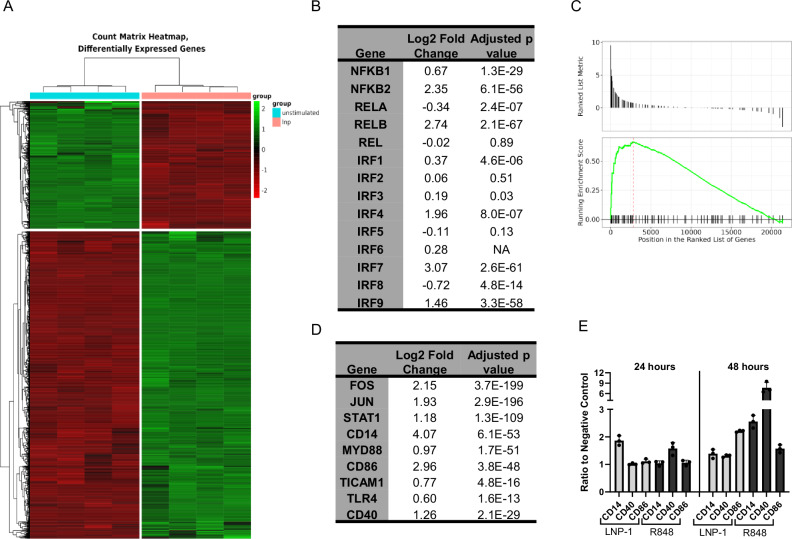
THP-1 transcriptional response to LNP-1. THP-1-Dual reporter cells were treated with LNP-1 (1 μg/mL) or media alone prior to RNA isolation and sequencing. Samples were sequenced in quadruplicate. Heatmap of 10,585 differentially expressed genes upon comparison of unstimulated to LNP-1-stimulated THP-1 cells out of a total of 57,773 genes detected (**A**). Table of NF-κB subunit and IRF genes with their corresponding log2-fold change and adjusted *p* values (**B**). Gene set enrichment analysis (GSEA) for KEGG gene set hsa04620Toll-like receptor signaling pathway (**C**). Table of selected genes from hsa04620Toll-like receptor signaling pathway (**D**). THP-1 cells were stimulated with LNP-1 (1 μg/mL), R848 (1 μg/mL) or media alone, and surface marker expression was measured after 24 or 48 hours by flow cytometry (*n* = 3). Data are shown as the ratio of mean fluorescence intensity (MFI) of the stimulated condition to the unstimulated negative control and represented as mean ± SEM (**E**). Adjusted p values were calculated by the Benjamini-Hochberg procedure.

Our data demonstrate that a monocyte cell line can be activated by empty ionizable LNP to induce NF-κB and IRF pathways, which induce cytokines and inflammatory profiles^38,42^. To define signaling cascades involved in the LNP-mediated activation, we analyzed the transcriptional response of THP-1 cells upon LNP stimulation. We chose LNP-1 as the model as it elicited the strongest activation of the tested LNPs. Comparing LNP-1 stimulated to unstimulated cells at 24 h, we identified 10,585 differential expressed genes (DEGs) out of 57,773 measured. We used a stringent cutoff criterion of a 0.01 false discovery rate and log 2-fold change (LFC) cutoff above 1 and below -1 to narrow down pathway analysis (Fig. 3A). Five genes encoding NF-κB subunits were measured and among those NFKB1, NFKB2 and RELB were significantly upregulated. Two met the DEG criteria, with NFKB1 just under the threshold. Evaluation of interferon regulatory factors demonstrated that IRF3, IRF4, IRF7, and IRF9 were upregulated. NF-κB activation initiates transcription of additional NF-κB subunits while IRF initiates a positive feedback loop through IFN signaling^43,44^. Thus, heightened expression of these transcripts is consistent with the reporter assay findings. Of the upregulated IRFs, only IRF4 and IRF7 met our criteria for DEG (Fig. 3B). IRF3 exhibited a small, but significant upregulation with a LFC of 0.19. The luciferase reporter gene in our THP-1 model is under the control of an ISG54 minimal promoter in conjunction with five IFN-stimulated response elements. Thus, all upregulated IRFs may contribute to the reporter response seen (Fig. 2A–C).

Using the list of DEGs, we identified gene sets and pathways in the gene ontology (GO), hallmark, and KEGG databases to understand the mechanisms that might be linked to ionizable LNP activation of immune responses. From the GO database, we identified 791 gene sets with 70 containing more than 50 DEGs (Supplementary Fig. 6A). We also identified thirteen and five differentially regulated pathways with the hallmark and KEGG databases, respectively (Supplementary Fig. 6B, C). Of the pathways identified, many were associated with immune activation, including cell death, interferon production, NF-κB-regulated cytokines, and TLR signaling (Supplementary Fig. 6A–C). The upregulation of interferon and NF-κB-regulated cytokine signaling pathways is consistent with our findings using the THP-1 reporter system.

We next utilized gene set enrichment analysis (GSEA), which considers all genes rather than only the DEGs (Fig. 3C). This analysis identified upregulation of the TLR Signaling pathway, indicating a mechanism of activation. Within this pathway, key genes involved in antigen presenting cell activation and T cell engagement were upregulated (Fig. 3D). We confirmed these findings at the protein level by measuring surface expression of CD14, CD40, and CD86 on THP-1 cells after stimulation with LNP-1 (Fig. 3D, E). As seen by flow cytometry, LNP-1 induced approximately 2-fold increases in CD14 and CD86 at peak expression levels and a 1.3-fold increase in CD40 (Fig. 3E). This data corresponds to the higher LFCs of CD14 and CD86, 4.07 and 2.96 respectively, with CD40 exhibiting an LFC of 1.26. In addition to NF-κB and IRF, other transcription factors are activated in response to LNP-1. JUN and FOS which encode AP-1 are both upregulated, suggestive of the crosstalk between signaling pathways (Supplementary Fig. 6A–C, Fig. 3D).

### TLR4 initiates signaling through MyD88 in response to ionizable LNPs

**Fig. 4:**
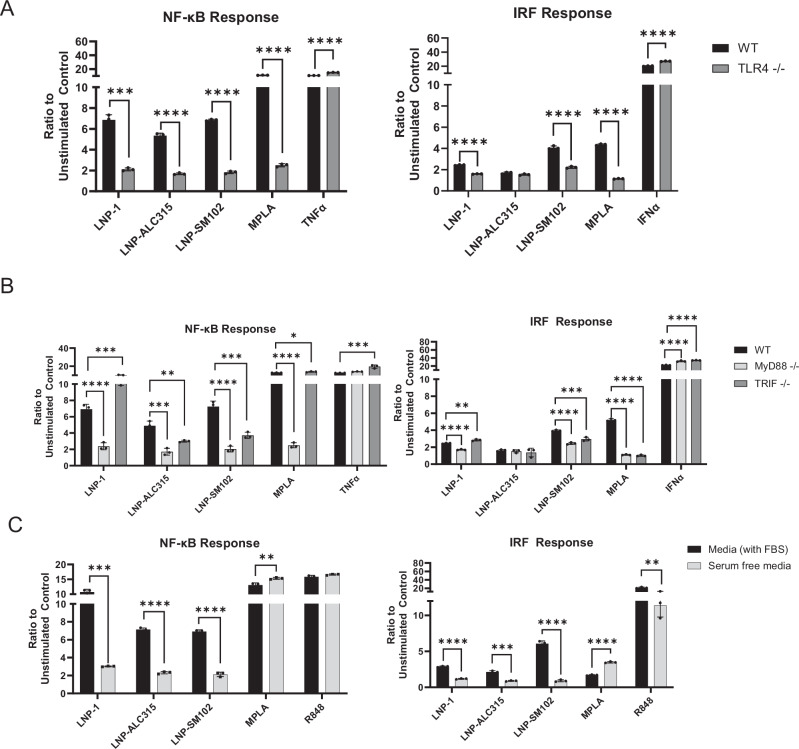
LNP-mediated NF-ĸB and IRF signaling transduction dependence on TLR4 and adaptor molecules. THP-1-Dual reporter cell lines were incubated for 72 h with LNP-1 (1 μg/mL), LNP-ALC315 (1 μg/mL), LNP-SM102 (1 μg/mL), MPLA (1 μg/mL), TNFα(50 pg/mL), R848 (1 μg/mL), IFNα(5 ng/mL) or media alone. NF-κB and IRF response were compared between wild-type (WT) and TLR4 knockout cell lines (**A**) and between the WT and MyD88 or TRIF knockout cell lines (**B**). THP-1 WT cells were incubated with stimuli in media containing serum (FBS) or without serum (**C**). The NF-ĸB and IRF responses were assessed and reported as a ratio of the reporter production of the stimulated condition to the media alone control (*n* = 3). Data are represented as mean ± SEM. Significance was assessed by *t-*test (A) or one-way ANOVA with Dunnett’s multiple comparisons test (**A,B**) or student’s *t*-test (**C**). *P ≤ 0.05, **P ≤ 0.01, ***P ≤ 0.001, ***P ≤ 0.0001.

Based on the upregulation of the TLR Signaling pathway in our transcriptomics data set and prior findings on cationic and one ionizable LNP, we hypothesized that TLR4 plays a key role in the ionizable LNP activation of monocytes^25,28,29,45^. We used the THP-1 reporter with the TLR4 gene knocked out to determine if THP-1 upregulation of NF-κB and IRF were driven by this signaling pathway (Supplementary Fig. 7). Upon comparing the THP-1 with the TLR4-KO, we measured a marked reduction in NF-κB and IRF responses to all ionizable LNPs (Fig. 4A) (Supplementary Fig. 8A). The loss of TLR4, however, did not completely abrogate the NF-κB or IRF activity (Fig. 4A) (Supplementary Fig. 8A). These findings suggest that TLR4 may be the principal receptor responsible for ionizable LNP-mediated activation of THP-1 cells, but other pathways may contribute. To ensure the accuracy of our data, we tested our LNP for presence of endotoxin, which can also trigger TLR4, and determined it was below the assay detection limit.

Additionally, TLR4 activation can be triggered by cell death^46^. There is a potential for LNP-mediated products of cell death to initiate TLR4 signaling detected in this study. However, a recent study demonstrated structural features of ionizable lipids of different LNP formulations engage TLR4 corroborating our findings with TLR4-KO and removal of the ionizable lipid. Together these finding support TLR4 signaling occurring separate from cell death^47^.

To further evaluate TLR4 involvement in sensing ionizable LNPs, we investigated adaptor proteins MyD88 and TRIF, which participate in the signaling cascade (Supplementary Fig. 7). Analysis of the transcriptomic data showed upregulation of MyD88 and TICAM1 genes, the latter encoding TRIF, in response to LNP-1 (Fig. 3D). Both NF-κB and IRF activation by all ionizable LNPs was reduced in MyD88 deficient THP-1 cells after 72 h (Fig. 4B) (Supplementary Fig. 8B). In contrast, TRIF deficient THP-1 cells showed a slight increase in NF-κB activation by LNP-1. LNP-ALC315 and LNP-SM102, however, elicited a reduced response in the TRIF-KO, albeit not to the same degree as with the MyD88-KO (Fig. 4B) (Supplementary Fig. 8B). Similar results were seen with the IRF response in the TRIF-KO. These results demonstrate that although all three LNPs are recognized by TLR4, they have varying reliance on the adaptor proteins though signaling was predominantly through the TLR4/MyD88 pathway.

To further investigate alternative pathways and evaluate the role of TLR4 in sensing the ionizable LNPs, we evaluated NF-κB and IRF activation using the A549 cell line with the same reporter constructs. The A549 cell line is derived from alveolar basal epithelial cells that do not express TLR4^48^. No NF-κB or IRF response to any LNP was observed (Supplementary Fig. 9) indicating the key role of TLR4 in sensing LNPs and failed to identify alternative pathways.

Next, we hypothesized that serum, facilitates LNP-TLR4 interaction and uptake, as has been shown with LPS^49^. To test if serum is required for LNP-mediated responses, we used serum-free conditions and demonstrated a significant decrease in both NF-κB and IRF activation (Fig. 4C, Supplementary Fig. 8C). Serum-free cultures exhibited reduced proliferation and unstimulated serum-free samples were used for normalization. These findings demonstrate ionizable LNPs rely on the presence of serum for THP-1 activation. To determine if TLR4 interaction also occurred in the endosome, we investigated colocalization of DiO-tagged LNP with TLR4 in early endosomes, as marked by Rab5a, which was not observed (Supplementary Fig. 8D). This data further suggests TLR4-LNP interaction occurs at the cell surface (Supplementary Fig. 7).

### Addition of mRNA does not enhance NF-κB or IRF responses induced by LNP

**Fig. 5:**
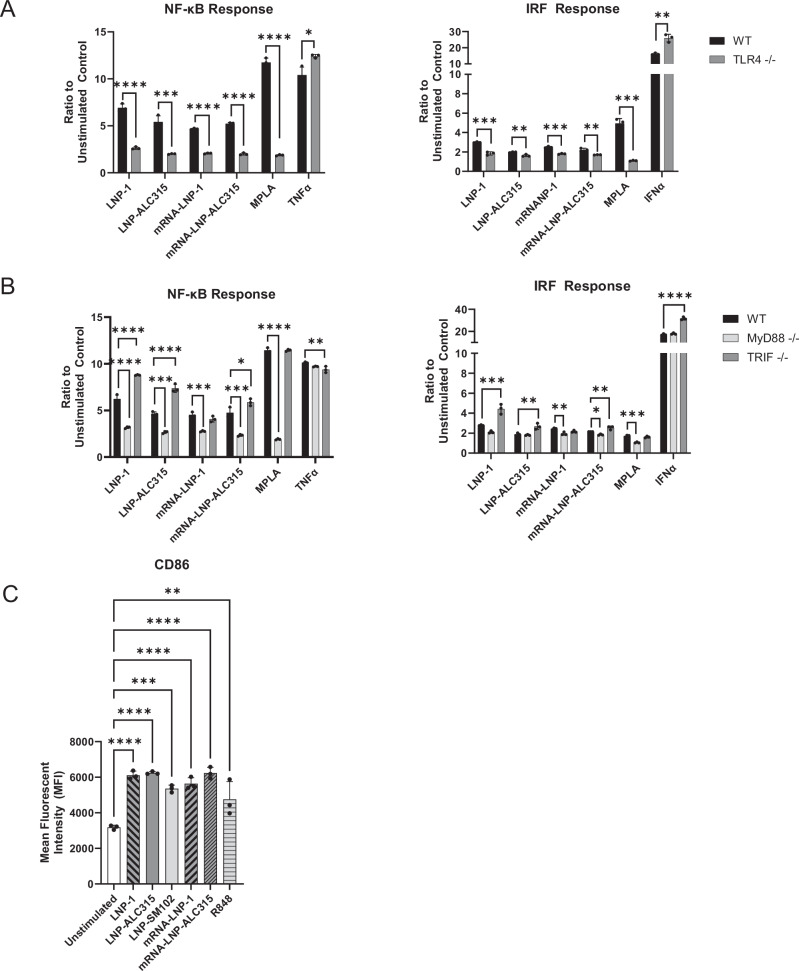
Response to LNPs compared to mRNA-LNP in THP-1. THP-1-Dual reporter cells were incubated with LNP-1 (1 μg/mL), LNP-ALC315 (1 μg/mL), LNP-SM102 (1 μg/mL), mRNA-LNP-1 (1 μg/mL), mRNA-LNPALC315 (1 μg/mL), MPLA (1 μg/mL), TNFα (50 pg/mL), IFNα (5 ng/mL), R848 (1 μg/mL), or media alone for 72 h. NF-κB and IRF response were compared between wild-type (WT) and TLR4 knockout cell lines (**A**) and between the WT and MyD88 or TRIF knockout cell lines (**B**). NF-ĸB and IRF responses were assessed and reported as a ratio of the reporter production of the stimulated condition to the media alone control (*n* = 3). THP-1 cells were stimulated and CD86 surface marker expression was measured after 72 h by flow cytometry as mean fluorescence intensity (MFI) (*n* = 3) (**C**). Data are represented as mean ± SEM. Significance was assessed using a one-way ANOVA with Tukey’s test for multiple comparisons. *P ≤ 0.05, **P ≤ 0.01, ***P ≤ 0.001, ****P ≤ 0.0001.

We next sought to determine the role of mRNA in innate activation induced by the BNT162b2 vaccine. Although modifications were made to the BNT162b2 mRNA component to reduce its immunostimulatory capacity^13–15^, we hypothesized that BNT162b2 mRNA vaccine would have heightened NF-κB and IRF activation compared to LNP alone. Surprisingly, our data showed that the response to mRNA-LNP was comparable to or lower than the corresponding empty LNP upon treatment of THP-1 reporter cells (Fig. 5A, B). R848 was previously shown to elicit a robust NF-κB and IRF activation (Fig. 1A, Fig. 2A), demonstrating a functional TLR7/8 response. Thus, the mRNA component did not augment the NF-κB and IRF signaling and did not stimulate additional pathways in the absence of TLR4, MyD88, or TRIF (Fig. 5A, B). Furthermore, CD86 expression on THP-1s was similar between mRNA-LNPs and corresponding empty LNP treated cells (Fig. 5C). Additionally, mRNA-LNP and empty LNP did not elicit NF-κB or IRF responses in A549s (Supplementary Fig. 9). These data suggest that ionizable LNPs are fully competent to activate a monocyte model of innate immunity in the absence of mRNA and that the addition of mRNA did not increase the response.

## Discussion

The advent of licensed mRNA vaccines marks a paradigm shift in immunization strategies, providing a platform which elicits robust adaptive immune responses and can be produced rapidly^4^. Interestingly, these vaccines lack a defined adjuvant, indicating intrinsic adjuvanticity. Though the precise mechanism remains incompletely understood, both the RNA component and LNP are implicated in induction of innate immunity^17,19,36^. Understanding the mechanism of mRNA vaccine adjuvanticity may provide insights for improving immunogenicity and/or reactogenicity of future vaccines. Therefore, the goal of this study was to better define the innate immune pathways activated by the empty ionizable LNPs, including those used in the BNT162b2 and mRNA-1273 mRNA vaccines.

Using a THP-1 monocyte cell line with reporter systems, we found that the three ionizable LNPs induce NF-κB and IRF activation to varying degrees. Activation was largely dependent on TLR4 through the MyD88 adaptor, with minimal TRIF dependence. Transcriptional profiling of LNP-1 stimulated THP-1 cells supported these findings, showing upregulation of TLR signaling. No NF-κB or IRF response occurred in the absence of the ionizable lipid component, indicating it is the driver of innate immune activation and suggesting structural differences in the ionizable lipids influenced signaling magnitude. Additionally, our data indicated signaling was primarily TLR4-MyD88 dependent, but additional pathways are likely induced by ionizable LNPs. Furthermore, activation required serum, but not endocytosis, supporting the role of surface expressed TLR4.

Notably, we observed that mRNA-containing LNPs did not increase IRF or NF-κB signaling in THP-1 cells compared to their empty LNPs, suggesting the LNP is the primary adjuvant in the BNT162b2 mRNA vaccine. While other pathways may be engaged, the predominant pathway utilized is via TLR4/MyD88 at the cell surface.

Responses varied across LNPs. LNP-SM102 (mRNA-1273 formulation) elicited the greatest IRF response, LNP-1 the strongest NF- κB response, and LNP-ALC315 (BNT162b2 formulation) showed similar NF-κB activation to LNP-SM102 and IRF activation similar to LNP-1. No response was observed without the ionizable lipid component, confirming its role as the driver of NF-κB and IRF activation. Although we did not test this with LNP-SM102, the ionizable lipid is likely the driver but we cannot rule out a role for its pegylated lipid. We posit that structural differences may influence strength of NF- κB and IRF activation, as seen with LPS variations across bacterial species in TLR4 signaling^50–52^. Thus, ionizable lipid design is a potential manner in which LNPs can be modified to enhance adjuvanticity or diminish reactogenicity.

Our findings on LNP-mediated innate immune activation are supported by Connors et al, who have demonstrated that dendritic cell and monocyte populations from PBMCs stimulated with LNP-1 exhibit TBK1 and IRF7 activation between 6 and 24 hours^17^. TBK1 activates both IRF3 and IRF7, and as our IRF reporter is produced upon activation of any IRF, IRF7 could be the source of reporter production seen in the THP-1s^53,54^. While, IRF3 is activated downstream of TLRs, such as TLR4^55–57^, our data did not show strong upregulation of IRF3 nor TRIF-dependence for LNP-1. However, IRF4 and IRF7 were both upregulated, suggesting they contribute to the response to LNP-1. As LNP-ALC315 and LNP-SM102 exhibited some TRIF dependence, IRF3 may play a role for these LNPs. Together, our data demonstrate that empty ionizable LNPs can elicit both NF-κB and IRF responses, albeit the definitive IRF activation requires further investigation.

Using THP-1 knockout lines, we found that cellular activation was predominantly dependent on TLR4-MyD88 signaling. Supporting our findings, an LNP with the MC3 ionizable lipid elicits reactogenicity in mice through the TLR4-MyD88 pathway, independent of TRIF^45^. Certain cationic LNPs can be detected by TLR4, and thus are able to stimulate innate immune responses^25,31^. Charge and structure of the lipid components appear to contribute to their stimulatory capabilities. Variations in head groups of cationic lipids have been demonstrated to modulate TLR4 signaling^28^. Moreover, a recent study highlighted the significance of amine head group structures in TLR4 interactions with ionizable lipids distinct from those investigated in this study^47^. Additionally, structural changes to hydrocarbon chain length as well as head group modifications could enhance or abrogate costimulatory molecule upregulation on murine dendritic cells^58^. Thus, structural variations can alter strength of signaling. However, despite structural differences in lipids, TLR4-MyD88 appears to be a common pathway for innate immune activation. Furthermore, no NF-κB and IRF responses were detected in A549s, which lack TLR4, further supporting TLR4 involvement in activation by ionizable LNPs.

Our data further demonstrates that serum proteins, are likely involved as serum was required for LNP-mediated activation for LNPs tested. These proteins may contribute to the formation of the protein corona surrounding the LNP, facilitating receptor-mediated uptake, or they may include the LPS-binding protein (LBP), which is essential for LPS engagement with TLR4^49,59^. However, further investigation is needed to clarify the specific mechanisms involved. The protein corona is particularly significant because its composition depends on the local environment of the LNP^59^. Consequently, intramuscular delivery of LNPs could result in a protein corona distinct from that of intravenously delivered LNPs. Therefore, understanding the role of the protein corona in LNP-mediated innate immune activation may require evaluation in specific biological contexts.

As neither the NF-κB nor IRF response was completely abrogated with absence of TLR4 or its adaptors, we suspect additional pathways contribute to detection of ionizable LNPs. Notably, a TLR4 knockout mouse model utilized in the study by Li et al. did not exhibit loss of vaccine responsiveness^36^. Others have shown a different ionizable LNP can elicit reactogenicity in mice through TLR4, suggesting that variations in activation strength and modeling may influence results^45^. Since our data indicates that LNP activation is not entirely dependent on TLR4 signaling, other pathways may provide sufficient stimulation in the absence of TLR4. Additionally, human and murine TLR signaling and expression are not fully consistent and may result in conflicting findings^60–63^.

Transcriptional profiling of cellular responses to LNP-1 showed upregulation of several pathways related to inflammation, cell death, and stress, which could provide insight into alternative methods of ionizable LNP recognition. Other adjuvants have been demonstrated to utilize those mechanisms for immune activation^12^. For instance, alum and saponin-based adjuvants induce cell stress^64–66^. Furthermore, vaccines that induce cell death have enhanced effectiveness^67^. As cationic LNPs have been observed to initiate inflammatory cell death, this is a plausible method for ionizable LNP immune activation and requires further investigation^27^.

The mechanisms by which ionizable LNPs activate THP-1 cells may also be linked to the delay in kinetics observed. During our investigation of the kinetics of the THP-1 response to the different LNPs, the peak of signaling occurred at 48 h. Although Connors et al. found that signaling of LNP-1 was highest at 24 h, they did not investigate later timepoints^17^. As MPLA (TLR4 agonist) and R848 (TLR7/8 agonist) elicited strong NF-κB responses within 24 h, we expected to see a more pronounced response to the LNPs at this time point. One explanation for the late onset activation observed may be that endocytosis is required. Since the cells are grown in media containing serum, a protein corona will form around the LNP^68^. This structure would sterically impede TLR4 detection until some level of breakdown occurs during endocytosis. Thus, we considered that the kinetics may be reflective of this requirement. Using a DiO-tagged LNP, we showed with flow cytometry and immunofluorescence microscopy that THP-1 cells take up LNP within 2 h and continuously over the 72 h measured. As this was suggestive of a threshold of uptake for LNP-mediated activation, we used different chemical inhibitors to block uptake. Only chloroquine elicited reduction in IRF response. Although the inhibitors impaired response to controls at early time points, they only minutely reduced LNP uptake for the first 24-hour period. Thus, it remains possible that signaling from the endosome occurs. However, TLR4 primarily utilizes MyD88 from the cell surface and switches to the TRIF-dependence after endocytosis^34,69^. Since we predominantly observed MyD88-dependence in the LNPs tested, this supports cell surface activation of TLR4. Additional investigation is needed to determine the mechanisms underlying the kinetics of ionizable LNP-mediated activation.

We took our study further to investigate the contribution of the mRNA component compared to the ionizable LNP alone. Although modifications have been made to the RNA to render it less immunostimulatory, there is still potential for detection of this RNA in some capacity by TLR7/8^13–15^. Additionally, any dsRNA remaining after mRNA purification may be detected by TLR3 or cytosolic PRRs such as MDA5. However, our data show that in the THP-1s, the NF-κB and IRF responses to mRNA-LNPs tested were similar to the responses to their corresponding empty LNP. Thus, the RNA component provided no immunostimulatory benefit in this model system. Our data contrast with findings suggesting that MDA5 is a key innate immune receptor for mRNA vaccination, with MDA5 knockout mice having markedly decreased innate and adaptive immune responses after vaccination with BNT162b2^36^. The vaccine used in that study was BNT162b2 created with GMP standards whereas our mRNA-containing LNPs were created with cellulose-purified mRNA, and thus could be responsible for the different results. Furthermore, in vivo MDA5 activation could come from dsRNA products of cell stress rather than dsRNA remnants from mRNA synthesis^70–72^.

Overall, our data show that three LNPs with structural variations in the ionizable lipid, are detected by TLR4 and activate NF-κB and IRF transcription factors in innate immune cells. TLR signaling is known to cause up-regulation of molecules involved in T-cell crosstalk and production of cytokines and TLR agonists have been utilized as adjuvants in clinical trials and licensed vaccines for such activation^73,74^. Our transcriptional data showing broad upregulation of molecules involved in cell activation pathways suggests that ionizable LNPs may be providing additional immune stimulation through, as of yet, unconfirmed pathways. Investigation into the mechanism behind TLR4 engagement may allow for the ability to adjust the lipid composition to balance tolerability with strength of stimulation. Additionally, our data indicates that the LNP, more so than RNA, is the predominant driver of mRNA vaccine-induced activation of THP-1 monocytes. Therefore, efforts could be focused on understanding how to create LNPs to target specific signaling pathways rather than further modification of mRNA to help guide vaccine and adjuvant design for safer, more effective, and potentially more durable mRNA vaccines.

## Methods

### Cell lines and culture

THP-1-Dual^TM^ a dual reporter human monocyte cell line (InvivoGen, San Diego, CA, USA) was obtained for use in reporter assays, microscopy, and flow cytometry. This suspension cell line stably expresses a secreted alkaline phosphatase (SEAP) reporter inducible by NF-κB and a secreted lucia luciferase reporter inducible by IRF pathway activation. Knockouts for MyD88, TRIF, and TLR4 in the dual-reporter background were also obtained from InvivoGen. THP-1 cells were grown in RPMI 1640 with 2 mM L-glutamine (Cytiva, Marlborough, MA, USA) supplemented with 25 mM HEPES (Thermo Fisher, Waltham, MA, USA), 10% heat-inactivated FBS, and Pen-Strep (100 U/ml–100 µg/ml). Cells were maintained at 37 °C with 5% CO_2_. Selection antibiotics, Blasticidin (10 µg/ml) and Zeocin (100 µg/ml) (InvivoGen, San Diego, CA, USA), were applied every other passage per manufacturer recommendation.

A549-Dual^TM^ a dual reporter human respiratory epithelial cell line (InvivoGen, San Diego, CA, USA) was obtained for use in reporter assays. These cells were grown in DMEM with 2mM L-glutamine (Thermo Fisher, Waltham, MA, USA) supplemented with 10% heat-inactivated FBS and Pen-Strep (100 U/ml-100 µg/ml). Cells were maintained at 37 °C with 5% CO_2_.

### mRNA preparation and lipid nanoparticle formulation

The diproline-modified SARS-CoV-2 Omicron variant spike glycoprotein (S2P) was codon optimized and cloned into an in vitro transcription template plasmid containing a T7 promoter, 5’ and 3’ UTR regions, and a 100 nucleotide poly(A) tail. mRNA was synthesized and co-transcriptionally capped using the Megascript Transcription kit (Thermo Fisher, Cat# AMB 1334) and the CleanCapTM dinucleotide system (Trilink Biotechnologies), precipitated, and purified using a modified cellulose-based chromatography method. Length and mRNA integrity were assessed using the Agilent Bioanalyzer 2100 system. Removal of double stranded RNA (dsRNA) contaminants was confirmed using dot blot, and endotoxin levels were measured using the Genscript ToxiSensor chromogenic assay (< 0.05 EU/mL). The S2P mRNA was stored frozen (1 mg/mL) at **−**20 °C in nuclease and pyrogen free water until use. mRNA was encapsulated into lipid nanoparticles as previously described (51). LNP lipid components were mixed in an ethanolic solution, and rapidly mixed with an aqueous phase containing the mRNA, dialyzed, and concentrated to 1 mg/mL, and stored at **−**80 °C until used (Supplementary Table 1, Supplementary Fig. 1).

LNP-ALC315, following the BNT162b2 formulation, was comprised of the ALC-0315 ionizable lipid, cholesterol, DSPC, and the ALC-0159 PEG-lipid. LNP-SM102, following the mRNA-1273 formulation, was comprised of the SM-102 ionizable lipid, cholesterol, DSPC, and PEG-DMG PEG-lipid. A third LNP formulation, designated as LNP-1, is proprietary to Acuitas Therapeutics. The proprietary lipid and LNP composition are described in US patent US10,221,127. LNP-1 is comprised of the a proprietary ionizable lipid, DSPC, cholesterol, and a PEG-lipid. LNP-1 was also tagged with the DiO fluorescent marker and designated as DiO-LNP. The fourth formulation made was an LNP, designated as LNP-no ionizable lipid, containing only cholesterol, DSPC, and the ALC-0159 PEG-lipid. LNPs were characterized for their hydrodynamic size, and polydispersity index (PDI), using dynamic light scattering (DLS), and the encapsulation efficiency and concentration measured using the RiboGreen RNA assay (Invitrogen, Cat# R11490). The empty LNP-ALC315 used was 54.07 nm with a PDI of 0.28 and zeta potential of -4.447. The empty LNP-SM102 used was 51.590 nm with a PDI of 0.1605 and zeta potential of -4.417. The LNP without the ionizable lipid was larger at 80.26 nm with a PDI of 0.05 and zeta potential of -8.548.

mRNA-LNPs were made with LNP-1 and LNP-ALC315, designated as mRNA-LNP-1 and mRNA-LNP-ALC315. The mRNA-LNP-ALC315 had an encapsulation efficiency of 98.17%, 66.4 nm diameter, 0.1090 PDI, and zeta potential of **−**11.55. LNP concentrations are given as the mRNA equivalent to enable comparison with the mRNA-LNP as the concentration of mRNA.

### Reporter assays

For both NF-κB and IRF reporter assays using the THP-1 cell line, cells were resuspended in RPMI 1640 with 2mM L-glutamine (Cytiva, Marlborough, MA, USA), 10% heat-inactivated FBS, and Pen-Strep (100 U/ml–100 µg/ml) at a concentration of 1 × 10^6^ cells/ml and 1 ml aliquoted per well of a 24-well plate. Reagents used individually to treat THP-1 cells included the LNP-1, LNP-ALC315, LNP-SM102, LNP-no ionizable lipid, mRNA-LNP-1, mRNA-LNP-ALC315, VacciGrade R848 (InvivoGen, San Diego, CA, USA), synthetic MPLA (InvivoGen, San Diego, CA, USA), IFNɑ (Miltenyi, Gaithersburg, MD, USA), LPS (Sigma-Aldrich, St. Louis, MO, USA), and TNFɑ (Thermo Fisher, Waltham, MA, USA). Empty LNPs and mRNA-LNPs were generously provided by the Alameh lab and University of Pennsylvania synthesized as described above^18^.

For analysis of endocytosis, cells were either left untreated or treated with the endocytosis inhibitors dynasore (50 μM, Fisher Scientific, Hampton, NH, USA), cytochalasin D (500 μM, Fisher Scientific, Hampton, NH, USA), chloroquine (50 μM, Fisher Scientific, Hampton, NH, USA) or methyl-β cyclodextrin (250 μM, Thermo Fisher, Waltham, MA, USA). THP-1s were treated two hours before addition of stimuli and every 24 h afterward. After thorough resuspension of reagent with THP-1, 200 µL of supernatant was removed at the indicated time points for measurement of reporter molecules. Cell counts were taken prior to removal of supernatant on each day to ensure similar growth curves. For the NF-κB reporter assay, 20 µL of supernatant was mixed with 180 µL of Quanti-Blue Solution (InvivoGen, San Diego, CA, USA) in a 96-well plate and incubated at 37 °C with 5% CO_2_ for 24 h. The absorbance at 600 nm was read using the Promega GlowMax plate reader (Promega, Madison, WI, USA). For the IRF reporter assay, 20 µL of supernatant was mixed with 50 µL of Quanti-Luc Solution (InvivoGen, San Diego, CA, USA) and the luminescence was immediately read at 0.1 s integration using the Promega GlowMax plate reader. Biological and technical replicates were performed for each condition in each assay.

For both NF-κB and IRF reporter assays using the A549 cell line, 6.25 × 10^3^ cells were seeded in each well of a 48 well plate with a final volume of 200 µL and incubated at 37 °C with 5% CO_2_ for 24 h. Supernatant was replaced with fresh media and stimulation was added. Cells were incubated for the designated time period and supernatant was removed for reporter assay as described above.

### Fluorescent microscopy

Cells were incubated with or without 1 µg/ml DiO-tagged LNP (DiO-LNP) at a concentration of 5 × 10^5^ cells/ml at 37 °C with 5% CO_2_ in 24 well plate. DiO-LNP was generously provided by the Alameh lab and the University of Pennsylvania synthesized as described above^18^. After incubation, cells were pelleted, washed, and resuspended in PBS. Cells were allowed to settle onto a coverslip at the bottom of 6-well plates for 30 min. Supernatant was gently removed, and cells were fixed with a 4% paraformaldehyde solution for 15 min. After fixation, cells were washed with PBS for two minutes twice. For intracellular staining, cells were permeabilized with saponin during fixation, blocked with 3% bovine serum albumin (BSA) (Miltenyi, Gaithersburg, MD, USA) for 1 h, and stained with anti-TLR4 antibody (anti-CD284) (Cat. No. 14-9917-82) (Thermo Fisher, Waltham, MA, USA) and anti-Rab5a antibody (Cat. No. 11947-1-AP) (Thermo Fisher, Waltham, MA, USA) for 1 h. Cells were subsequently washed with additional BSA and stained with secondary antibodies; anti-mouse IgG Alexa Fluor555 (Cat No. 4409S) (Cell Signaling Technology, Danvers, MA, USA) and anti-rabbit IgG Alexa Fluor647 (Cat No. 4414S) (Cell Signaling Technology, Danvers, MA, USA) for 1 h. Then, cells were stained with 1 µg/ml DAPI (BioLegend, San Diego, CA, USA) for 10 min in the dark and subsequently washed. Coverslips were mounted using ProLong Gold antifade reagent (Invitrogen, Waltham, MA, USA) and imaged on the Zeiss700 confocal microscope (Zeiss, Jena, Germany) using the ZEN 2.3 SP1 software.

### Flow cytometry

THP-1 cells at a concentration of 1 × 10^6^ cells/ml were incubated with DiO-LNP (1 µg/ml), LNP-1 (1 µg/ml), LNP-ALC315 (1 µg/ml), LNP-SM102 (1 µg/ml), mRNA-LNP-1 (1 µg/ml), mRNA-LNP-ALC315 (1 µg/ml), VacciGrade R848 (1 µg/ml) (InvivoGen, San Diego, CA, USA), or media alone at 37 °C with 5% CO_2_. For surface marker investigation, cells were washed with PBS and stained with Human TruStain FcX (BioLegend, San Diego, CA, USA) at 4 °C for 5 min to prevent nonspecific binding followed by surface staining with antibodies listed in Supplementary Table 2 at 4 °C for 20 min. Cells were fixed with a 4% paraformaldehyde solution for 15 min. For assessing kinetics of DiO-LNP uptake, cells were immediately washed and fixed. Cells were run on the Cytek Aurora Spectral Flow Cytometer and analyzed using FlowJo v10.

### Sequencing

THP-1 cells at a concentration of 1 × 10^6^ cells/ml were incubated with or without LNP-1 at 1 µg/ml for 24 h at 37 °C prior to RNA extraction with Qiagen RNeasy Plus Mini Kit (Qiagen, Hilden, Germany). Each condition was set up in quadruplicate with 1 million cells per sample. RNA was quantified using Qubit RNA Broad Range Assay Kit (Thermo Fisher, Waltham, MA, USA). For all samples, a total RNA amount of >100 ng was used as input for library preparation using the TruSeq Stranded total RNA Library Preparation Kit (Illumina, San Diego, CA, USA) using IDT for Illumina – TruSeq RNA UD Indexes. Sequencing libraries were quantified by real-time PCR using the KAPA Library Quantification Complete kit (Roche, Basel, Switzerland) and assessed for size distribution and absence of free adapters and adapter dimers on a Fragment Analyzer. Sequencing libraries were pooled and quantified by real-time PCR as above, clustered and sequenced on an Illumina NovaSeq 6000 using a Reagent Kit v1.5 (200 cycles) with run parameters generating paired-end reads at 100 bp length.

### Sequencing analysis

Fastq files obtained from the sequencing run were initially processed through the nf-core rnaseq pipeline via NextFlow using recommended pipeline settings^75,76^. The pipeline includes FASTQC, Trim Galore, SortMeRNA, STAR, featureCounts, StringTie, Salmon, Qualimap, dupRadar, Preseq, and RSeQC. Reads were aligned to the GRCh37 reference genome. Subsequent analysis was performed with R 4.3.2 using Jupyter Notebooks and the Tidyverse (v2.0.0) package. We used DESeq2 (v1.42.0) to identify differentially expressed genes (DEGs) between unstimulated and LNP-1-stimulated samples with 4 samples in each group. The Benjamini-Hochberg procedure was used for multiple testing correction. Additionally, we implemented a false discovery rate of 0.01 and a cutoff of a logfold change above 1 or below -1. Then, we performed logfold change shrinkage using apeglm (v1.24.0)^77^ and matched gene IDs to gene symbols to create a table of DEGs. The table of DEGs was run through Human MSigDB Collections (v7.5.1): hallmark gene sets (H), KEGG (C2: curated gene sets), and the GO (C5: ontology gene sets - GO: Gene Ontology gene sets). Gene Set Enrichment Analysis (GSEA) was performed using the Bioconducter clusterProfiler (v4.8.3) package.

### Statistical analysis

Statistical analyses were performed using GraphPad PRISM (v10, GraphPad Software Inc., Boston, MA, USA) using a one-way ANOVA followed by Dunnet’s test for multiple comparisons to a single control or by Tukey’s test for multiple comparisons between all groups. Results are expressed as means with standard error of the mean (SEM). Values of *P* < 0.05 were considered statistically significant. For transcriptomics data, the Benjamini-Hochberg procedure was implemented during determination of DEGs and pathway enrichment analysis to correct for multiple comparisons. The adjusted *p*-values were reported for these results. Significance for GSEA was determined by comparing the enrichment score to a null distribution through permutation testing.

## Supplementary information

Supplementary Information

## Data Availability

Data will be made available upon request. Sequencing data is available in the NCBI Sequence Read Archive under reference number PRJNA1196969.
