# Supplementary material for: Ionizable lipid nanoparticles of mRNA vaccines elicit NF-κB and IRF responses through toll-like receptor 4
Source: NPJ Vaccines. 2025 Apr 17;10:73. doi: 10.1038/s41541-025-01124-x (PMC12006303; doi:10.1038/s41541-025-01124-x)
Supplement: Supplementary file 1 — Supplementary Information [file 41541_2025_1124_MOESM1_ESM.pdf]

	**LNP-1**	**LNP-315**	**LNP-SM102**	**LNP no ionizable lipid**
Ionizable lipid	*Proprietary*	ALC-315	SM-102	-
Helper lipids	*Proprietary PEG-lipid*<br>DSPC<br>Cholesterol	ALC-0159<br>DSPC<br>Cholesterol	PEG-DMG<br>DSPC<br>Cholesterol	ALC-0159<br>DSPC<br>Cholesterol
Molar ratio<br><br>(ionizable lipid : PEG-lipid : Cholesterol : DSPC)	*Proprietary*	46.3:<br>1.6:<br>42.7:<br>9.4	50:<br>1.5:<br>38.5:<br>10	0:<br>1.6:<br>42.7:<br>55.7

Supplemental Table 1. Composition of lipid nanoparticles utilized.

Marker	Fluorochrome	Clone	Manufacturer	Cat. No.
HLA DR	BUV395	G46-6	BD	564040
L/D	L/D blue	-	Thermo Fisher	L34962A
CD70	BUV737	Ki-24	BD	612856
CD86	PE-Cy5	IT2.2	BioLegend	305408
CD80	PE-Cy7	2D10	BioLegend	305218
CD14	APC Cy7	M5E2	BioLegend	301820

Supplemental Table 2. Antibody Panel for Spectral Flow Cytometry. Antibodies were titrated before use in experiments.

A

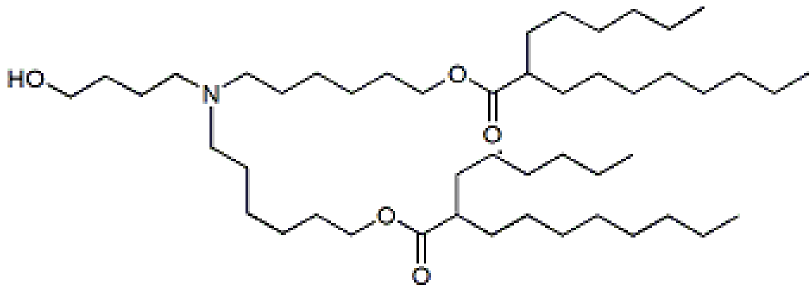

ALC-315

B

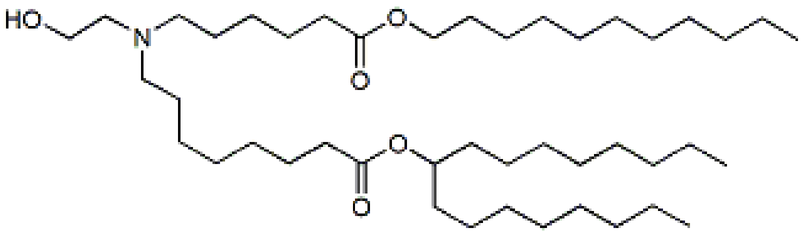

SM-102

Supplemental Figure 1: Structures of ionizable lipid components. Structures of the ionizable lipid ALC-315 in the BNT162b2 mRNA vaccine and LNP-ALC315 (A), and SM-102 in the mRNA-1273 mRNA vaccine and LNP-SM102 (B).

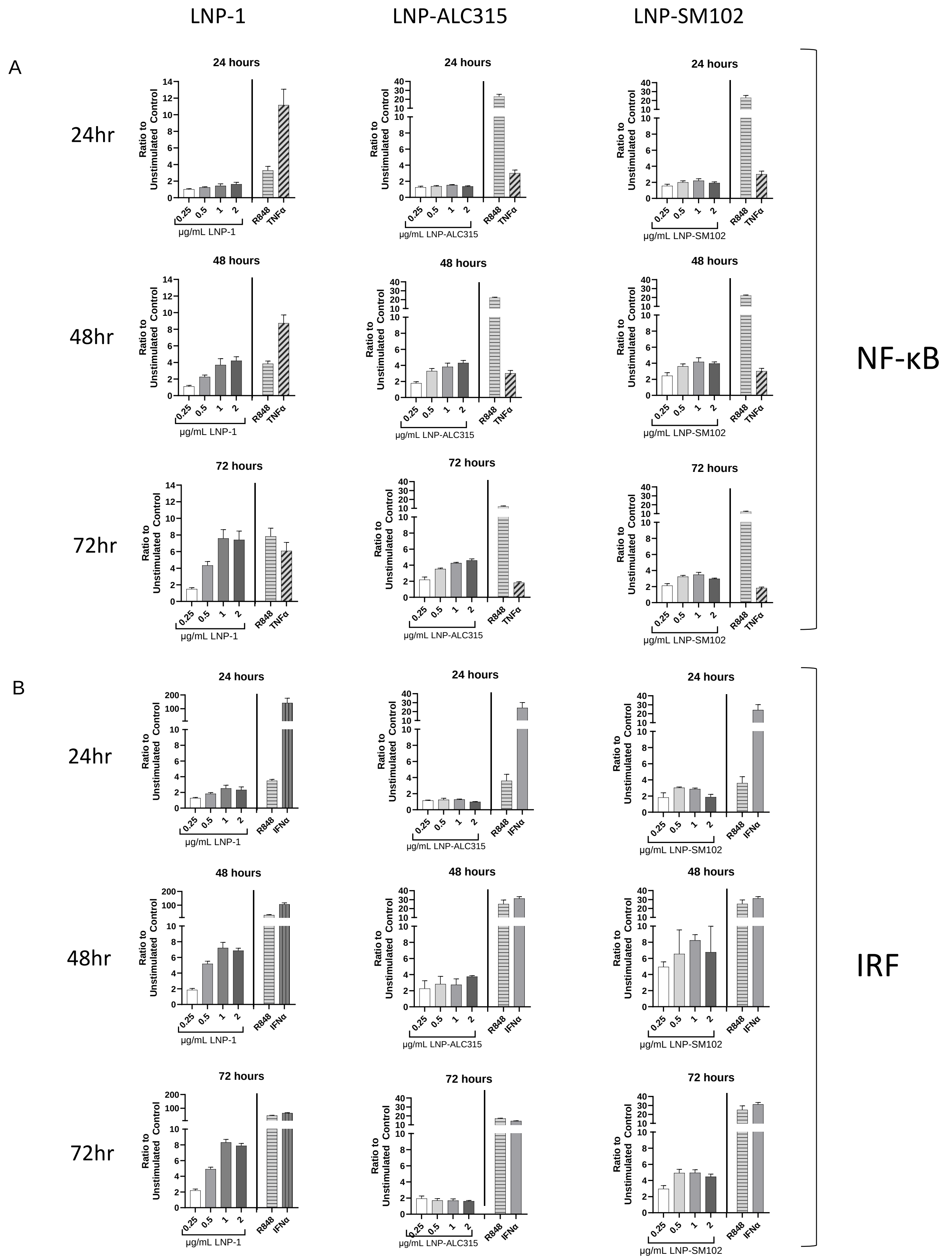

Supplemental Figure 2. Dose dependent response to LNP-1, -ALC315, and -SM102. THP-1-Dual reporter cells were incubated with LNP-1 (0.25-1 µg/mL), LNP-ALC315 (0.25-1 µg/mL), LNP-SM102 (0.25-1 µg/mL), R848 (1 µg/mL), TNFα (50 pg/mL), IFNα (5 ng/mL), or media alone for the indicated time duration, and reporter production was measured from the supernatant for either NF-κB (A) or IRF (B) (*n* = 3). The NF-κB and IRF responses were assessed and reported as a ratio of the reporter production of the stimulated condition to the media alone control. Data are represented as mean ± SEM.

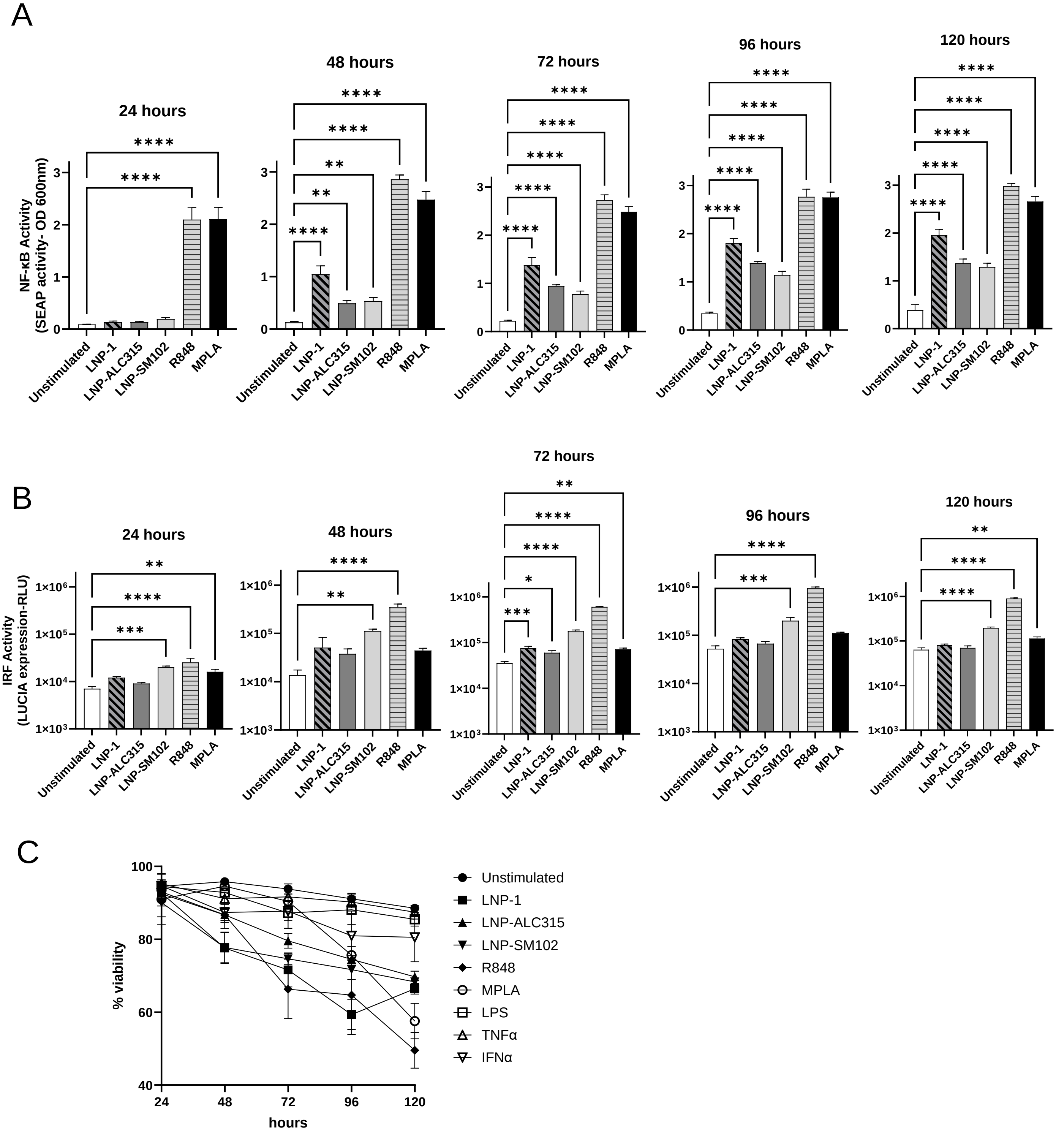

Supplemental Figure 3. Signaling transduction in THP-1 reporter cell line. THP-1-Dual reporter cells were incubated with LNP-1 (1 µg/mL), LNP-ALC315 (1 µg/mL), LNP-SM102 (1 µg/mL), R848 (1 µg/mL), TNFα (50 pg/mL), IFNα (5 ng/mL), or media alone for the indicated time duration, and reporter production was measured from the supernatant for either NF-κB (A) or IRF (B) (*n* = 3). Cell viabilities were recorded for each stimulation condition over 120 hours (*n* = 3) (C). Data are represented as mean ± SEM. Significance was assessed using a one-way ANOVA with Dunnett's test for multiple comparisons. *=P≤0.05, **= P≤0.01, ***= P≤0.001, ****= P≤0.0001

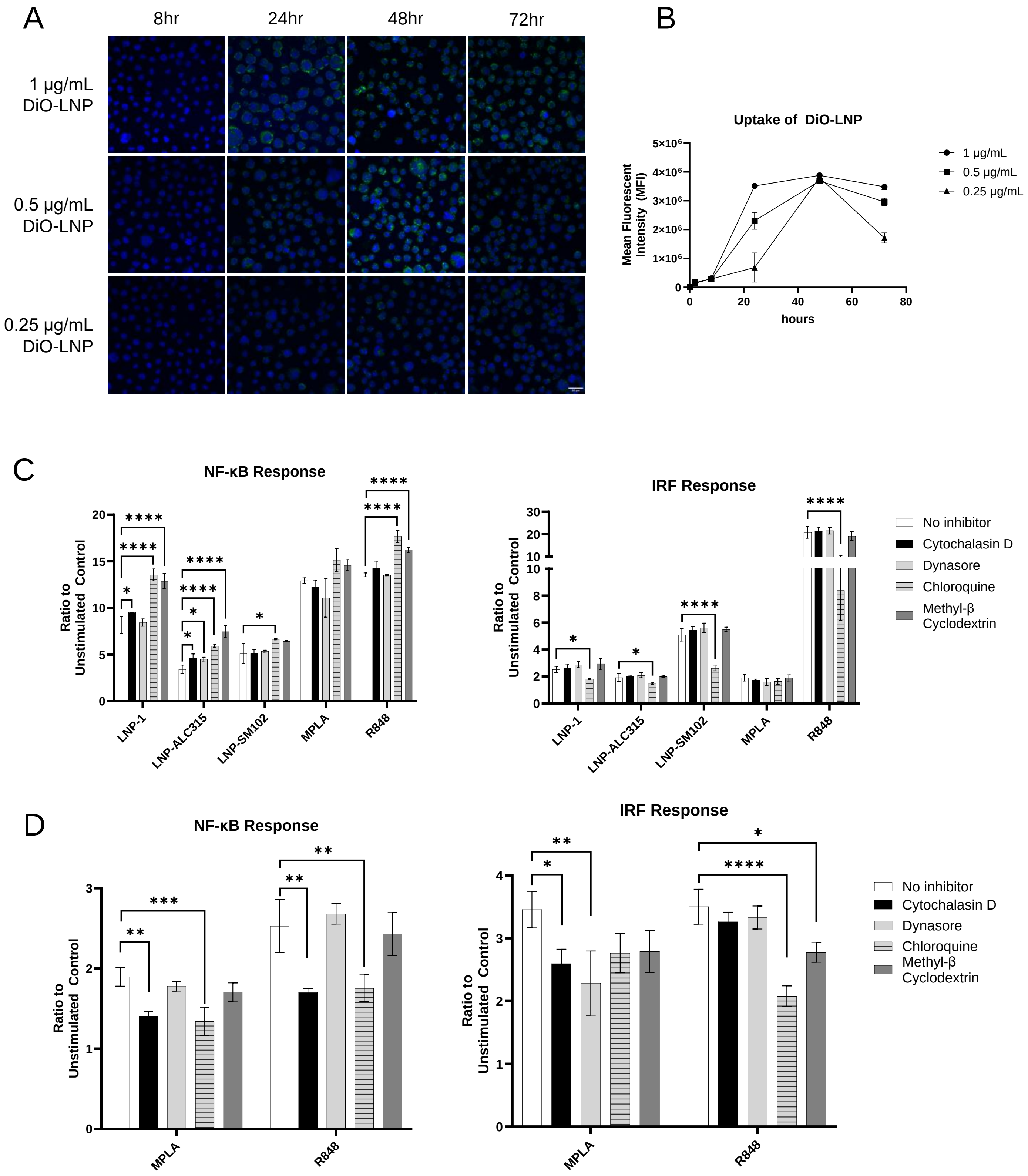

Supplemental Figure 4: THP-1 uptake of LNP. Images of DiO-LNP uptake by THPs, representative images. DAPI was used as a counterstain. Scale bar indicates 20 µm (A). Uptake of DiO-LNP (median fluorescence intensity, MFI) over time (*n* = 3) (B). THP-1-Dual reporter cells were incubated for 72hr (C), 6hr (D left), or 24hr (D right) with cytochalasin D (500 µM), dynasore (50 µM), chloroquine (50 µM), or methyl-β cyclodextrin (250 µM) for 2 hours prior to addition of the stimuli and cells were treated every 24 hours after. Reporter production was measured in the supernatant for either NF-κB or IRF (*n* = 3) (C,D). Data are represented as mean ± SEM. Significance was assessed using a one-way ANOVA with Dunnett's test for multiple comparisons. *=P≤0.05, **= P≤0.01, ***= P≤0.001, ****= P≤0.0001

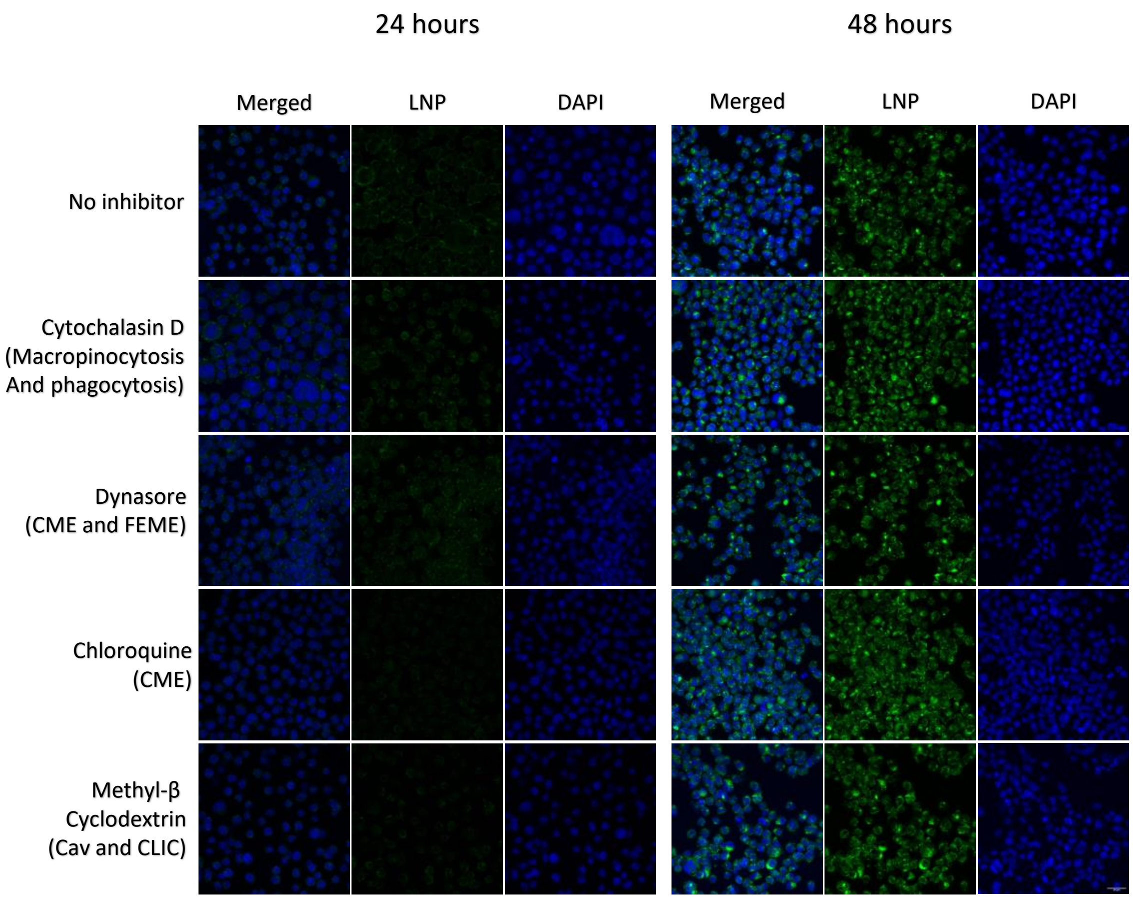

Supplemental Figure 5: THP-1 uptake of LNP with endocytosis inhibitors. Images of DiO-LNP uptake by THP-1 Dual reporter cells treated with endocytosis inhibitors cytochalasin D (500 μM), dynasore (50 μM), chloroquine (50 μM) or methyl-β cyclodextrin (250 μM) for 2 hours prior to addition of the stimuli and cells were treated every 24 hours after. Images were taken at 24 and 48 hours post-incubation with DiO-LNP-307, representative images. DAPI was used as a counterstain. Scale bar indicates 20 μm. (CME – clathrin-coated pit-mediated endocytosis, FEME – fast endophilin-mediated endocytosis, Cav – caveolae, CLIC – clathrin-independent carrier endocytosis)

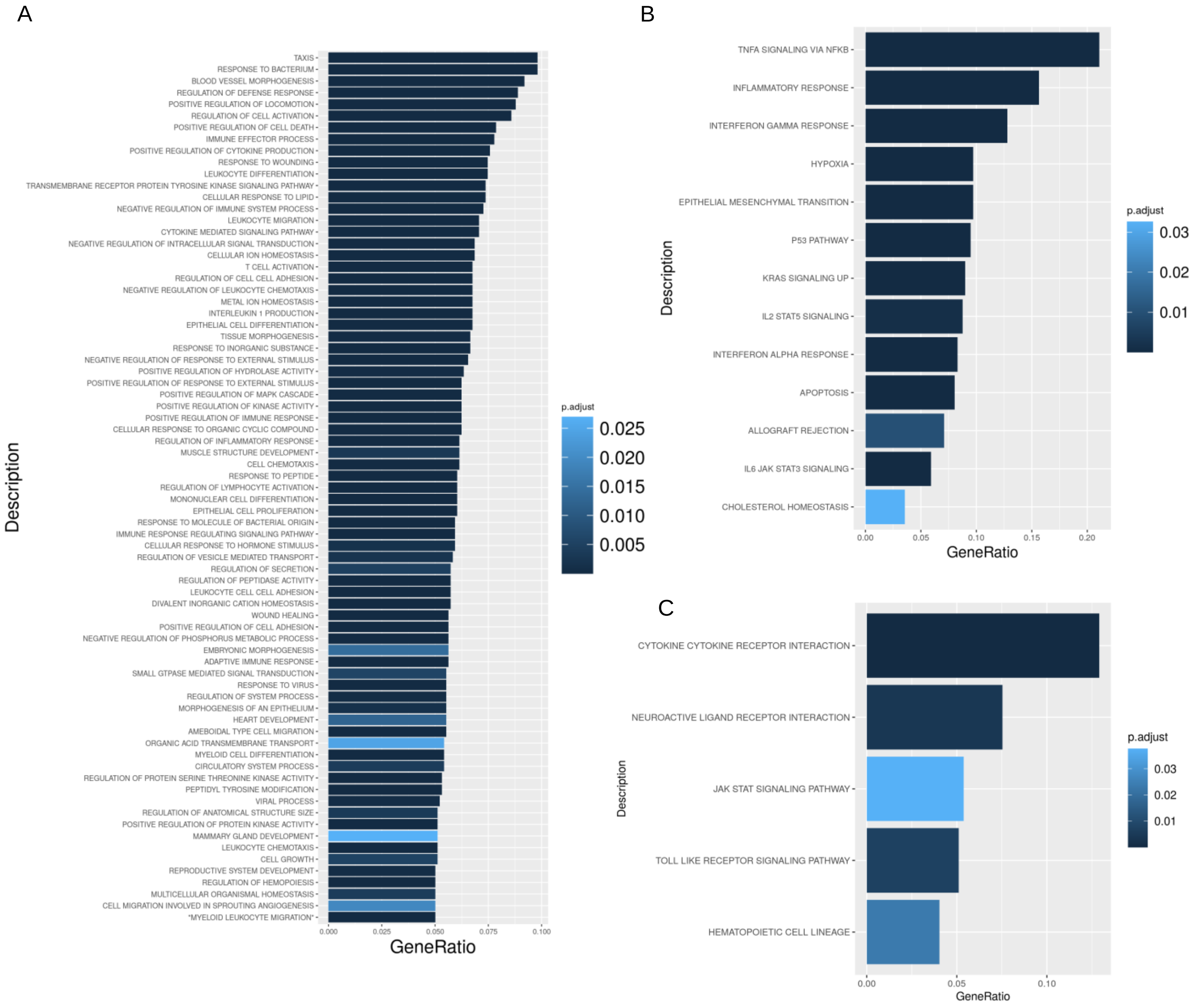

Supplemental Figure 6. Over-representation analysis of THP-1 response to LNP-1. Over-representation analysis is shown using the Gene Ontology (A), Hallmark (B), and KEGG (C) databases by ratio of differentially expressed genes in the designated pathway to the total number of genes in that pathway. Differentially expressed genes were defined as those with a log2 fold change above 1 or below -1, using a false discovery rate cutoff of 0.01.

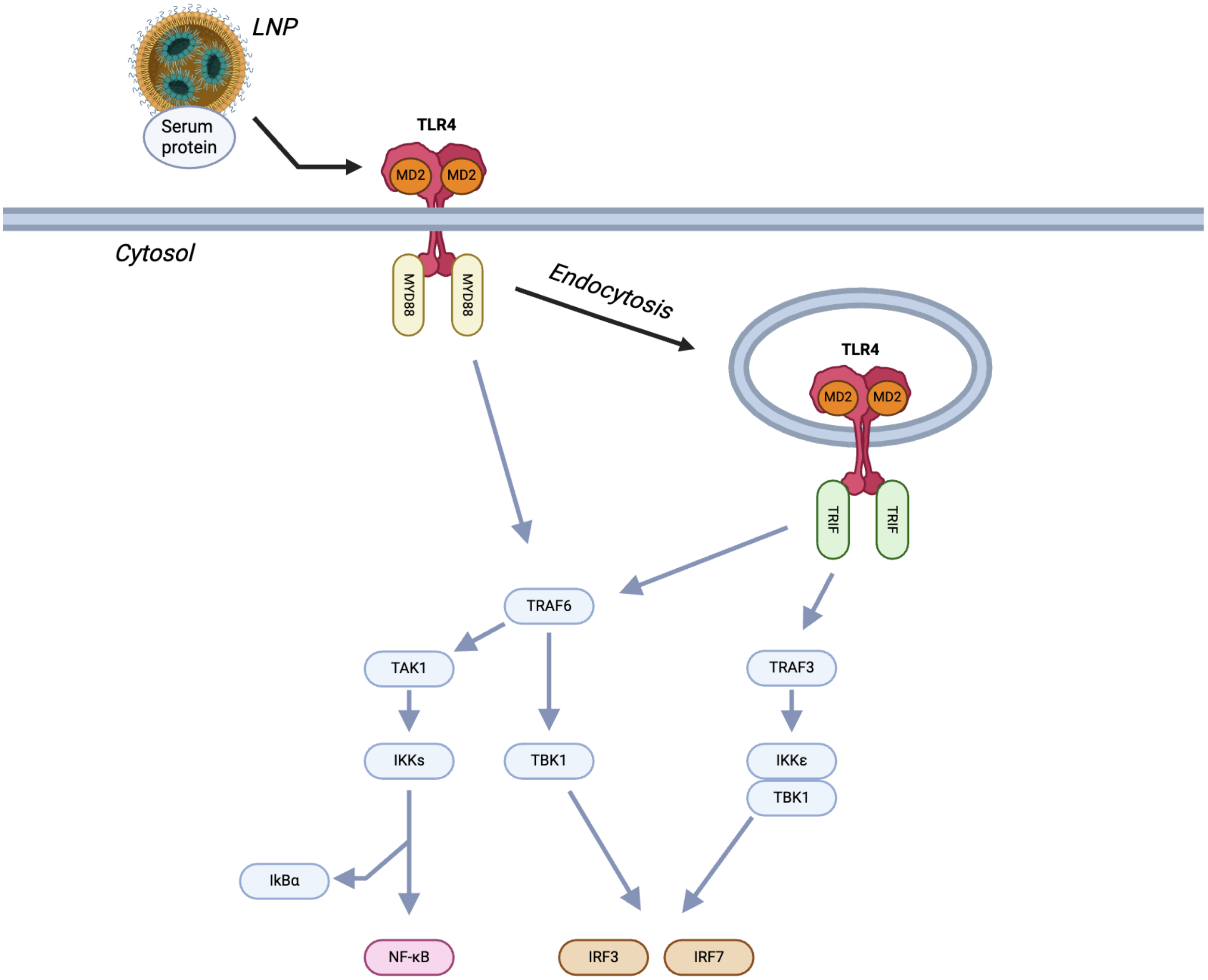

Supplemental Figure 7: NF-κB and IRF signaling cascade. TLR4 signaling pathway, simplified. Created in BioRender.com.

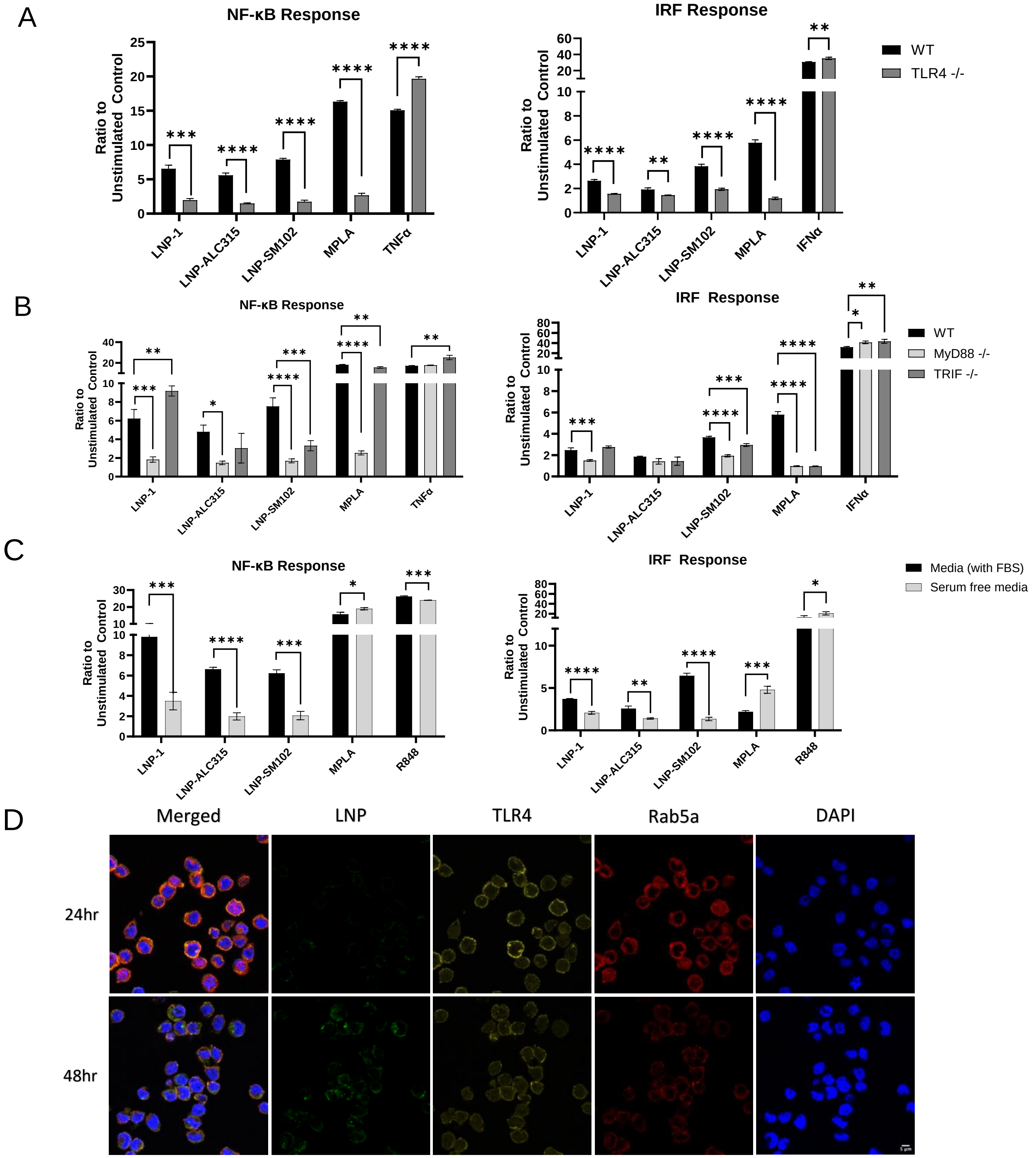

Supplemental Figure 8. LNP-mediated NF-κB and IRF signaling transduction dependence on TLR4 and adaptor molecules. THP-1-Dual reporter cell lines were incubated for 48 hours with LNP-1 (1 µg/mL), LNP-ALC315 (1 µg/mL), LNP-SM102 (1 µg/mL), MPLA (1 µg/mL), TNFα (50 pg/mL), R848 (1 µg/mL), IFNα (5 ng/mL) or media alone. NF-κB and IRF response were compared between wild-type (WT) and TLR4 knockout cell lines (A) and between the WT and MyD88 or TRIF knockout cell lines (B). THP-1 WT cells were incubated with stimuli in media containing serum (FBS) or without serum (C). The NF-κB and IRF responses were assessed and reported as a ratio of the reporter production of the stimulated condition to the media alone control (*n* = 3). Images of DiO-LNP-1 uptake by THPs, representative images. Cells were stained for TLR4 and the Rab5a early endosome marker. DAPI was used as a counterstain. Scale bar indicates 5 µm (D). Data are represented as mean ± SEM (A-C). Significance was assessed by t-test (A,C) or one-way ANOVA with Dunnett's multiple comparisons test (B). *= P≤0.05, ** = P≤0.01, ***= P≤0.001, ****= P≤0.0001

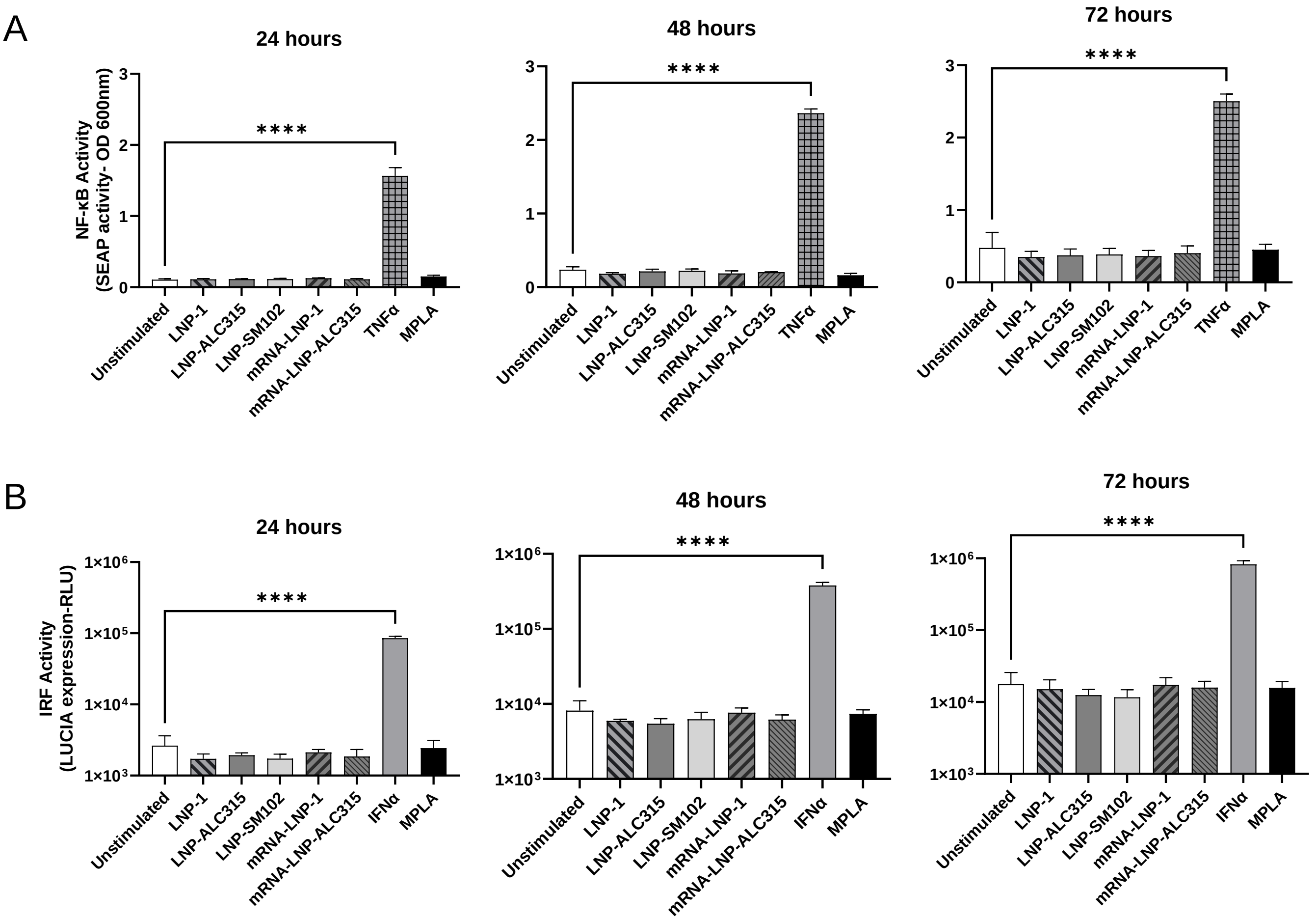

Supplemental Figure 9: A549 response to empty LNP and mRNA-LNP. A549-Dual reporter cells were incubated with LNP-1 (1 µg/mL), LNP-ALC315 (1 µg/mL), LNP-SM102 (1 µg/mL), mRNA-LNP-1 (1 µg/mL), mRNA-LNP-ALC315 (1 µg/mL), TNFα (100 ng/mL), IFNα (5 ng/mL), MPLA (1 µg/mL), or media alone for the respective time points, and supernatant was assayed for presence of reporter for either NF-κB (A) or IRF activation (B) (n = 3). Data is represented in respective reporter measurement units. Data are represented as mean ± SEM. Significance was assessed using a one-way ANOVA with Dunnett's test for multiple comparisons. *=P≤0.05, **= P≤0.01, ***= P≤0.001, ****= P≤0.0001
